# Correspondence on: “Neurolymphatic clearance in neurodegenerative disease: Emerging mechanisms and potential translational strategies”

**DOI:** 10.1016/j.jpra.2026.02.030

**Published:** 2026-03-06

**Authors:** Christophe Al Sammour, Nathan De Lissnyder, Vanessa Marron Mendes, Michela Schettino

**Affiliations:** a Université Libre de Bruxelles – Belgium, Lenniksebaan 808, 1070 Brussels; b Department of Plastic and Reconstructive Surgery, CHIREC Braine l’Alleud Hospital, Wayez Street 35, 1420 Braine-l’Alleud, Belgium; c Department of Neurosurgery, UZ Brussel, Brussels, Belgium; d Vrije Universiteit Brussel – Brussels, Belgium

*Dear Sir*,

The review by Kappos et al.^1^ on neurolymphatic clearance in neurodegenerative diseases provides a comprehensive synthesis of the anatomy, physiology, and emerging translational strategies targeting the neurolymphatic system, and successfully highlight the potential role of lymphatic modulation in conditions such as Alzheimer’s and Parkinson’s disease.

Beyond neurodegeneration, the concept of neurolymphatic dysfunction may find a particularly robust and clinically accessible application in chronic subdural hematoma (CSDH). While the review appropriately focuses on neurodegenerative disorders, extending this conceptual framework to CSDH may offer unique methodological and direct clinical applicability.

A major limitation in evaluating neurolymphatic modulation in neurodegenerative diseases lies in the irreversible nature of the underlying pathology. Cortical atrophy, neuronal loss, and established structural damage cannot regress, even if lymphatic clearance were to be enhanced. Furthermore, clinical outcomes rely largely on indirect measures, such as cognitive scales, which are influenced by multiple confounders and evolve slowly over time. This makes it inherently difficult to establish a direct and objective relationship between neurolymphatic modulation and clinical benefit.

In contrast, chronic subdural hematoma represents a potentially reversible pathological state. It is characterized by fluid accumulation, chronic inflammation, and membrane formation, without primary neuronal destruction. Improvements in clearance mechanisms may therefore translate into measurable reductions in hematoma volume, resolution of mass effect, and functional recovery.^2^ Importantly, outcomes in CSDH are readily quantifiable using imaging-based metrics, recurrence rates, and need for re-intervention, providing objective and reproducible endpoints rarely achievable in neurodegenerative conditions. It should be acknowledged that current evidence does not establish neurolymphatic dysfunction as a primary causal mechanism in chronic subdural hematoma, but rather suggests a potentially contributory pathway within a complex inflammatory and vascular process.

Emerging experimental and clinical evidence further supports a neurolymphatic contribution to CSDH pathophysiology.^3^ Meningeal lymphatic vessels have been proposed to contribute in the clearance of subdural collections, and modulation of their function appears to influence hematoma resolution. Notably, recent data suggest that vitamin D supplementation and medical treatment with statins may accelerate chronic subdural hematoma resorption, potentially through effects on meningeal lymphatic vessel structure and function and/or broader anti-inflammatory or vascular mechanisms.^4^ While not a therapeutic recommendation, these findings provide a proof of concept that pathways involved in hematoma clearance may be biologically modulated.

From a surgical perspective, framing CSDH as influenced by localized neurolymphatic dysfunction may also offer new insights. Chronic inflammation, membrane persistence, and recurrence share conceptual and pathophysiological similarities, at least at a conceptual level, with mechanisms described in peripheral chronic lymphoedema.^5^ This analogy may help refine surgical timing, drainage strategies, and adjunctive approaches aimed at reducing recurrence. Moreover, CSDH constitutes a clinically relevant interface between neurosurgery and lymphatic reconstructive principles, aligning well with the translational focus highlighted by Kappos et al.

Therefore, chronic subdural hematoma could serve as a valuable human model in which neurolymphatic modulation could be explored using volumetric imaging, recurrence rates, and re-intervention as objective endpoints, and in a manner directly relevant to surgical practice. Exploring neurolymphatic concepts in this context may not only strengthen the biological plausibility of lymphatic modulation but also accelerate meaningful clinical translation.

We congratulate the authors on their important contribution and hope that this perspective may further stimulate interdisciplinary discussion on the broader clinical relevance of neurolymphatic clearance.

## Sources of funding

The authors received no financial support for the research, authorship, and publication of this article.

## Declaration of competing interest

The authors declare that there are no relevant conflicts of interest.
